# Stay Tuned: What Is Special About Not Shifting Attention?

**DOI:** 10.1371/journal.pone.0016829

**Published:** 2011-03-14

**Authors:** Durk Talsma, Jonne J. Sikkens, Jan Theeuwes

**Affiliations:** 1 Department of Cognitive Psychology, Vrije Universiteit Amsterdam, Amsterdam, The Netherlands; 2 Department of Experimental Psychology, Ghent University, Ghent, Belgium; Rikagaku Kenkyūsho Brain Science Institute, Japan

## Abstract

**Background:**

When studying attentional orienting processes, brain activity elicited by symbolic cue is usually compared to a neutral condition in which no information is provided about the upcoming target location. It is generally assumed that when a neutral cue is provided, participants do not shift their attention. The present study sought to validate this assumption. We further investigated whether anticipated task demands had an impact on brain activity related to processing symbolic cues.

**Methodology/Principal Findings:**

Two experiments were conducted, during which event-related potentials were elicited by symbolic cues that instructed participants to shift their attention to a particular location on a computer screen. In Experiment 1, attention shift-inducing cues were compared to non-informative cues, while in both conditions participants were required to detect target stimuli that were subsequently presented at peripheral locations. In Experiment 2, a non-ambiguous “stay-central” cue that explicitly required participants not to shift their attention was used instead. In the latter case, target stimuli that followed a stay-central cue were also presented at a central location. Both experiments revealed enlarged early latency contralateral ERP components to shift-inducing cues compared to those elicited by either non-informative (exp. 1) or stay-central cues (exp. 2). In addition, cueing effects were modulated by the anticipated difficulty of the upcoming target, particularly so in Experiment 2. A positive difference, predominantly over the posterior contralateral scalp areas, could be observed for stay-central cues, especially for those predicting that the upcoming target would be easy. This effect was not present for non-informative cues.

**Conclusions/Significance:**

We interpret our result in terms of a more rapid engagement of attention occurring in the presence of a more predictive instruction (i.e. stay-central easy target). Our results indicate that the human brain is capable of very rapidly identifying the difference between different types of instructions.

## Introduction

The human mind is capable of selecting and holding sensory information that is task-relevant and discarding what is not relevant. This is accomplished through a set of processes that are collectively known as attention [1]. Several studies have found evidence for the existence of an attentional control network that is predominantly located in frontal and parietal brain areas [2], [3], [4], [5], [6]. This so-called fronto-parietal network is thought to send biasing signals to the perceptual brain areas. These biasing signals then result in a selective change in sensitivity of neurons that are responsive to certain stimulus features over others [7], [8], [9], [10].

Shifts in attention are often induced by a symbolic cue presented in the center of the display instructing participants to voluntarily shift their attention to one of two lateral locations on a computer screen (cf. [11]). Following the presentation of the cue, an imperative stimulus that requires a response is presented at the cued or at the uncued location. A common method to track the time course of an attention shift involves the analysis of event-related brain potentials (ERPs) that are time-locked to the onset of the cue. Initially, ERPs elicited by leftward and rightward pointing cues were compared against each other. These studies revealed a number of positive and negative ERP components that were found over the hemisphere contralateral to the direction indicated by the cue. Of the components that were originally reported in the literature, the early-directing attention negativity (EDAN) [12] was observed as a negatively shifted waveform over the posterior areas contralateral to the location indicated by the cue, at a latency of about 200–300 ms after cue onset. While this component was originally thought to reflect attentional control operations, presumably representing a change in neural sensitivity of the perceptual brain areas, more recent evidence suggested that the EDAN may reflect attentional processing of the cue itself [13]. A second component, the anterior-directing attention negativity (ADAN) [14], [15], has been thought to reflect a generic, modality unspecific, attentional control mechanism [14], [16], [17]. Although the ADAN is still predominantly interpreted as a component that is related to attentional orienting, recent studies have either questioned this interpretation [18], [19], [20] or alternatively attributed functionality beyond attentional orienting to the ADAN [21], [22].

Although the comparison of ERPs elicited by leftward and rightward shifts of attention has provided many valuable insights into the dynamics of attentional shifts, it has been argued that this comparison is rather limited, because it can only isolate those brain processes that are related to the specific direction of orientation [23]. In order to isolate the full set of processes involved in shifting attention, one needs to compare a shift condition (regardless of the direction of the shift) with a neutral baseline condition that is equal to the shift condition, except that there are no attention shifts. Talsma et al. [23] compared ERP responses to left and right pointing arrow symbols against a non-informative cue, consisting of a white bar, and concluded that this comparison yields a much larger set of activations than the traditional left vs. right comparisons. These activations included an initial positivity (∼100–380 ms after cue onset) that seem to correspond to a sequence of frontal and parietal activations (subsequently labeled as “Shift-Related Positivity [24]), and a later sustained negativity that was predominantly observed when the interval between cue and imperative stimulus was relatively long. It should be noted, however, that one should exercise caution in interpreting results that involve a “neutral” baseline, because differences in strategy may confound possible experimental effects [25].

Consequently, it cannot be ruled out that completely that task-demands and cognitive strategies may affect the processes that are evoked by a non-informative cue. It was concluded [23] that the shift-related positivity observed in relation to their attention-directing cues bore some resemblance to earlier findings reported in the literature [26], [27], but with the noted exception that the shift-related positivities in the latter two studies were characterized by a more posterior distribution and/or later onset latencies. Therefore, it is possible that the much lower stimulus onset asynchronies (SOAs) between cue and target may have resulted in a higher demand on the prefrontal areas.

**Figure 1:**
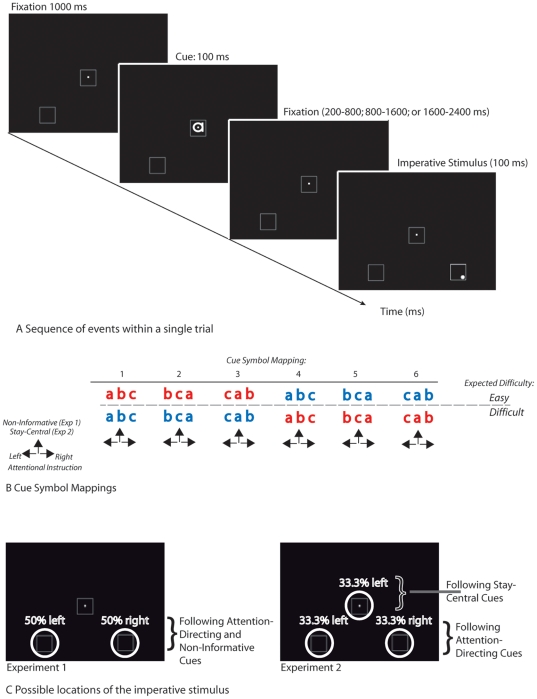
Schematic layout of the present paradigm. A) Sequence of events within a single trial. Following an initial 1000 ms fixation screen, a symbolic cue was presented with a 100 ms duration. Cues consisted of lower case letters a, b, and, c. that were printed in URW Gothic font. After a variable interval, an imperative stimulus was presented consisting of the transient (100 ms) brightening of one of the two peripheral boxes. In Experiment 2, the central box would brighten when preceded by a stay-central cue. Occasionally a faint gray dot was presented inside one of the corners of the box. This dot served as a target stimulus. b) Schematic depiction of the cue symbols and their functional meaning within the experiment; see main text for details. c) Highlight of the possible locations where the target stimuli could be presented in each experiment.

**Figure 2:**
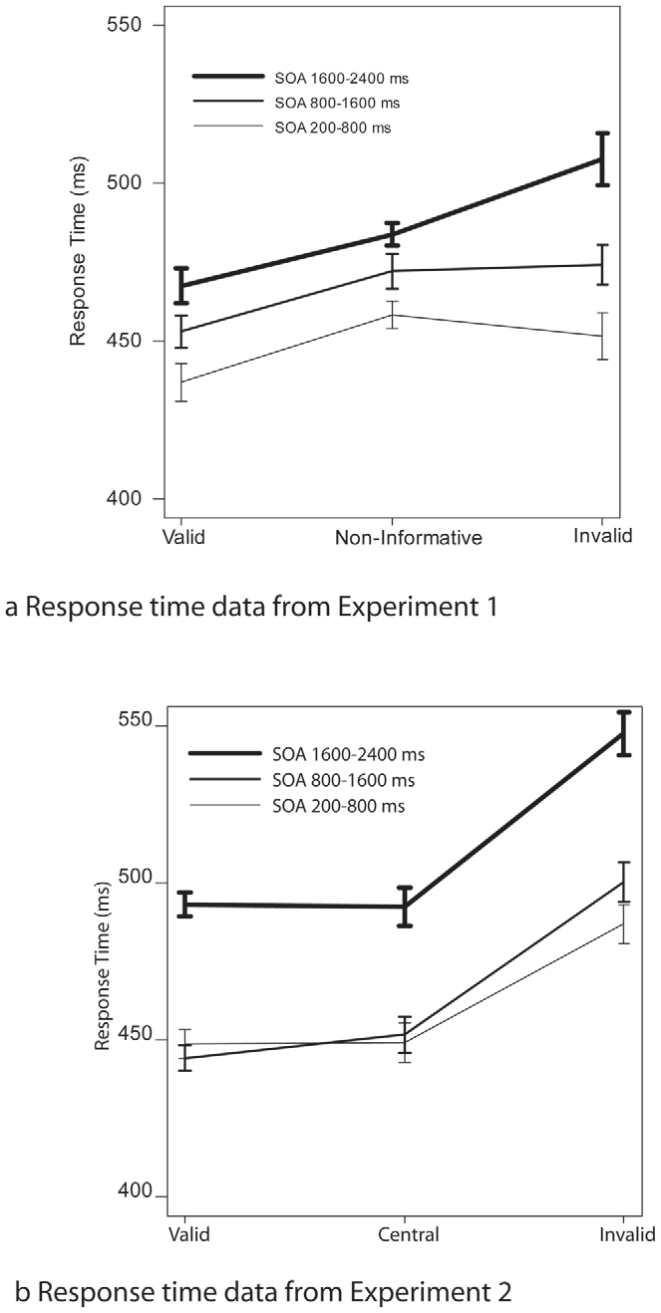
Mean Response times and hit-rates. a) results from Experiment 1. At intermediate and long SOAs between cue and target stimuli, a classical cueing effect can be observed, whereas at shorter SOAs, responses to targets that were preceded by a non-informative cue took somewhat longer to process that those preceded by either a valid or an invalid cue. Hit rates did not differ significantly between conditions. b) results from experiment 2. Note that response times and accuracies to the validly cued peripheral targets and those to the centrally cued targets did not differ significantly from each other, but that responses in these two conditions were faster and more accurate than those to invalidly cued peripheral targets.

Here we report two experiments, using variations of a symbolic cueing paradigm [11], that set out to investigate the concerns addressed above (see Figure 1). In experiment 1, we used a traditional non-informative cue and compared ERPs elicited by this neutral cue with ERPs elicited by attention shift-inducing cues. In contrast, in experiment 2, we replaced the non-informative cue by a cue that explicitly instructed participants to stay focused at the central location (“stay-central” cue; Figure 2c). Importantly, as symbolic cues we used letters, in which the cue-symbol mapping was counterbalanced across participants (Figure 2b). Since this rules out the possibility that the differences between attention-directing and non-informative cues were confounded by physical differences, this design also allowed us to study the effects of possible early latency attention shift-related components on the ERPs. If a non-informative cue resulted in participants not shifting attention, we would expect to find similar cue-elicited ERP waveforms elicited by a stay-central cue as we would expect to find for a non-informative cue.

In addition, we cued the difficulty of the possible target stimulus in advance. If an increase in anticipated difficulty would correlate with a stronger focus of attention, we would expect that cues that predicted more difficult targets would elicit a stronger attention effect. Finally, we systematically varied the SOA between cue and target to investigate possible effects of time pressure on the attentional system. We expected that if time-pressure is a mediating factor in frontal lobe activation, we would find more and earlier frontal activation in the shorter SOA conditions than in the longer SOA conditions.

## Results

### 2.1 Experiment 1

The goal of this experiment was to investigate the time-course of the processes involved in attentional orienting, as revealed by comparing ERP activity elicited by attention shift-inducing cues against a non-informative, “neutral” baseline condition. In addition, we investigated the effects of task demands on the attentional orienting processes by manipulating the SOA between the cue and the subsequent imperative stimulus, as well by pre-cueing the difficulty of the imperative stimulus. To this end, a symbolic cueing paradigm was used in which a centrally placed letter indicated the location that needed to be attended. In addition, the color of the cue letter indicated whether participants should selectively prepare for an easy or a hard target, which allowed us to explore the possible contributions of selective preparation for an easy or difficult stimulus to detect.

### 2.2 Results

#### 2.2.1 Behavioral Data

Mean response times to correctly responded target stimuli are shown in Figure 2a. In this analysis, target difficulty was not considered, because for the behavioral measures this factor was confounded with a physical difference in stimuli. This factor will be of concern for the ERP analyses described below. Response times increased with increasing SOAs, as indicated by a significant main effect of SOA (*F*(2,30) = 12.65; *p*<.00001). In addition, a significant cueing effect was found (*F*(2,30) = 4.79; *p*<.05). The exact pattern of the cueing effect differed across SOA conditions, however, as expressed in a significant interaction between Cue Type, and SOA (F(4,60) = 2.87; p<.05). As shown in Figure 2a, the cueing benefits, as expressed as the response time difference between validly and non-informatively cued trials remained constant across SOA condition. In contrast, the cueing costs, as expressed as the response time difference between non-informatively, and invalidly cued trials differed across SOA conditions. Post-hoc tests indeed yielded no significant interaction between Cue Type and SOA for the valid vs. non-informative comparison (F<1), whereas a significant interaction these factors was present for non-informative vs. invalid comparison (F(2,30) = 5.05; p<.05). Post-hoc tests indicated that in the long SOA conditions, response times to non-informatively cued targets were longer than response times to validly cued targets t(15) = 3.6; p<.01), and shorter than response times to invalidly cued targets t(15) = 2.07; p<.05). For the intermediate and short SOA conditions, response times to non-informatively cued targets were significantly longer than response times to validly cued targets (t(15) = 2.28; p<.05), and t(15) = 2.89; p<.05) respectively). For the latter two conditions, response times did not differ significantly between non-informatively and invalidly cued targets (t<1 for both comparisons). Mean hit rate was about 74%. No statistically significant differences were observed on the accuracy measures.

#### 2.2.2 ERP data

**Figure 3:**
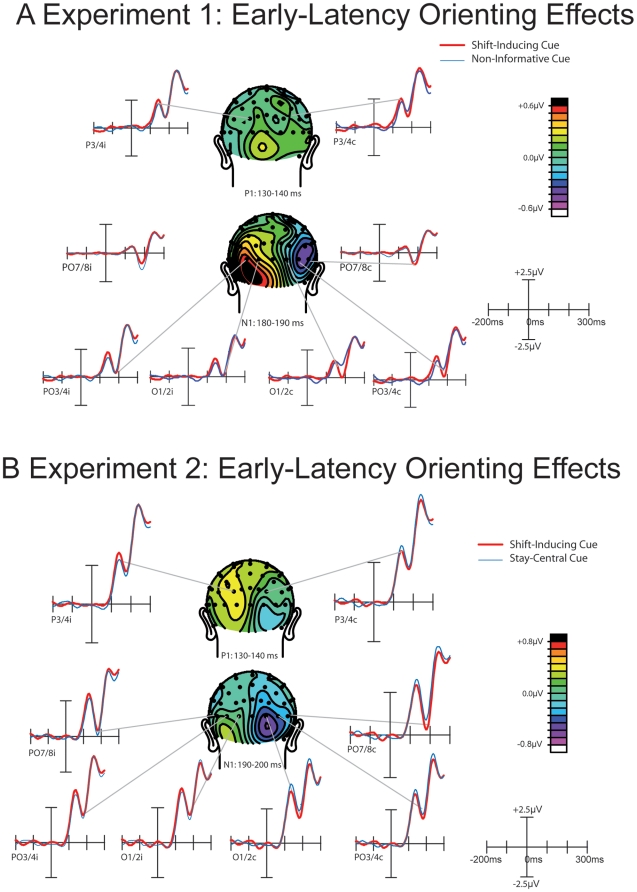
Early-latency cueing effects. a) results from Experiment 1. An occipitally distributed N1 component, elicited by shift-inducing cues, was larger over the contralateral hemisphere and smaller over the ipsilateral hemisphere, compared to the N1 elicited by non-informative cues. a) results from Experiment 2. In addition to replicating the N1 attention effect, we also observed a significant P1 modulation here that preceded the N1 in time. This P1 effect was found mainly over the ipsilateral hemisphere and is characterized by a more parietal scalp distribution than the subsequent N1.

**Figure 4:**
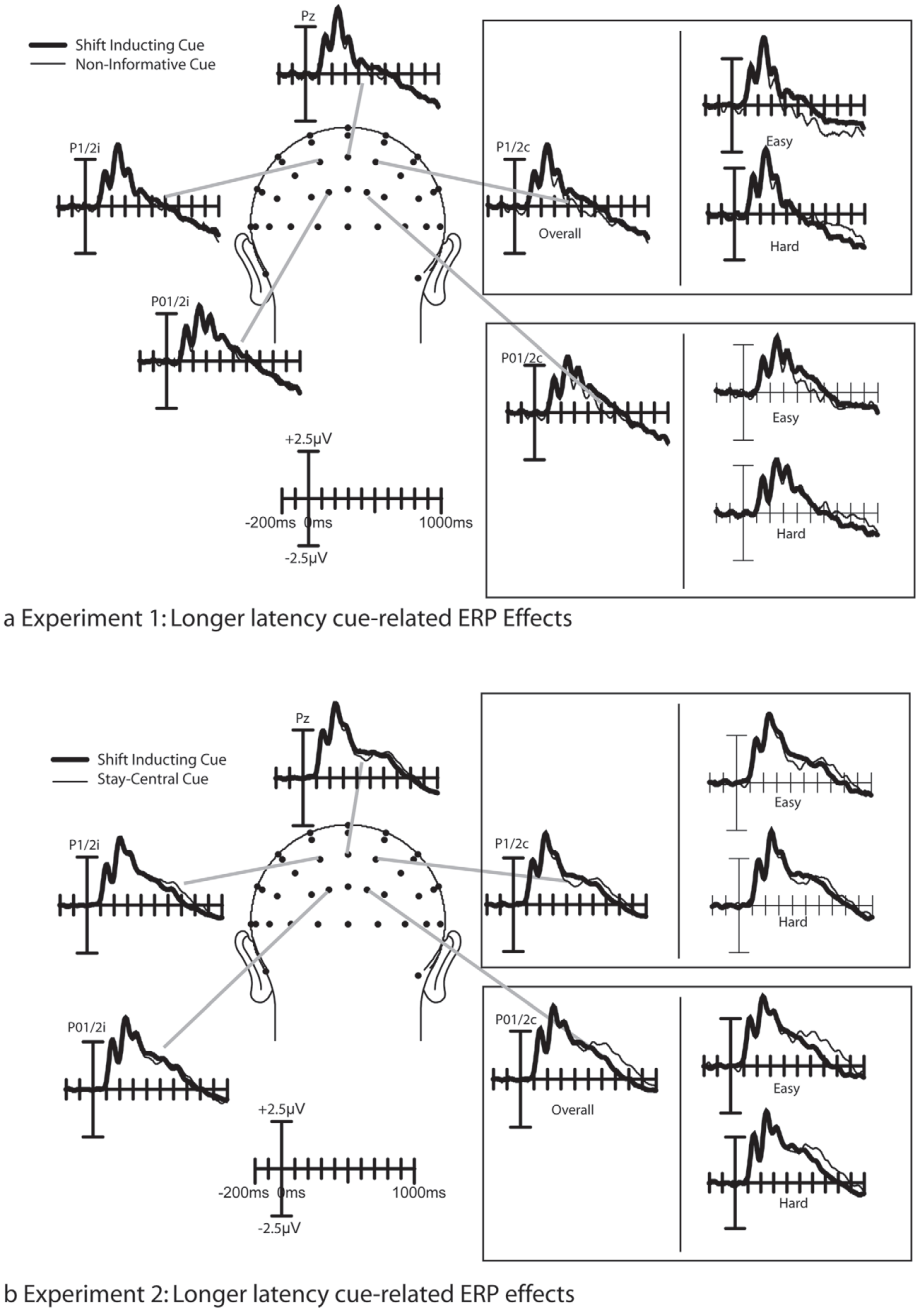
Longer latency cueing effects. a) results from Experiment 1. From about 200 to 400 ms after cue-onset, the ERPs elicited by shift inducing cues were positively shifted, relative to the ERPs elicited by non-informative cues. At longer latencies, a clear distinction between cues predicting and easy target and cues predicting a difficult target can be seen; whereas for cues predicting an easy target the ERP wave form was more positive for shift inducing cues, whereas this was not the case for cues predicting a difficult target. This effect can in particular be observed over the contralateral hemisphere. b) results from experiment 2. Two effects can be noted here. First, an early latency cueing effect was present for cues predicting an easy target. At longer latencies, the ERPs elicited by stay-central cues are positively displaced, starting at around 500 ms after cue onset, whereas no such positive displacement can be observed for shift-inducing cues. Notably, this effect was larger for cues predicting an easy target than for cues predicting a difficult target.

As shown in Figures 3a and 4a, the cue symbols elicited a well-established sequence of ERP components. Over parietal and occipital areas, P1 and N1 components were observed, which were followed by a more broadly distributed series of three positive peaks that is characteristic for a symbolic cue [23], [24], which have been labeled as the shift-related positivity. These peaks, in turn, are followed by a sustained negative going potential at longer latencies.

Although both the contralateral P1 and the N1 component appeared to be larger in response to a shift-inducing cue than to a non-informative cue, only the contralateral N1 amplitude effect was significant as a three way interaction between Cue Type, Hemisphere, and Electrode (*F*(2,24) = 5.39; *p*<.05; *GG* = .61). This interaction indicates that the contralateral N1 elicited by shift-inducing cues was significantly more negative than the contralateral N1 elicited by non-informative cues, and that the scalp distribution of this contralateral amplitude enhancement was characterized by a relatively sharp focus. No such interactions could be observed for the P1 effect (*F*(2,24) = 1).

Finally, Figure 4a suggests the presence of an early negative deflection at posterior electrodes P1, Pz, P2, PO1, POz, and PO2, at around 80 ms after cue onset. No main effect of Cue Type was found in this latency range, however, a trend toward significance could be observed for the interaction between Cue Type and Difficulty (F(1,15) = 3.57; p<.08) in this latency range.

As shown in Figure 4a, significant cueing effects could be observed at longer latencies. Following the more lateral N1 effect described above, a medially distributed shift-related positivity could be observed. Figure 4a suggests that this effect is most pronounced over P1/2 and PO1/2 electrodes, it can be seen that the shift-inducing cues are positively displaced, relative to the non-informative cues. This effect was statistically significant as an interaction between Cue Type and Laterality, between 280 and 340 ms after stimulus onset (Fs(1,15) = 4.98–6.85; ps<.05), and between 440 and 520 ms (Fs(1,15) = 3.38–5.33).

**Figure 5:**
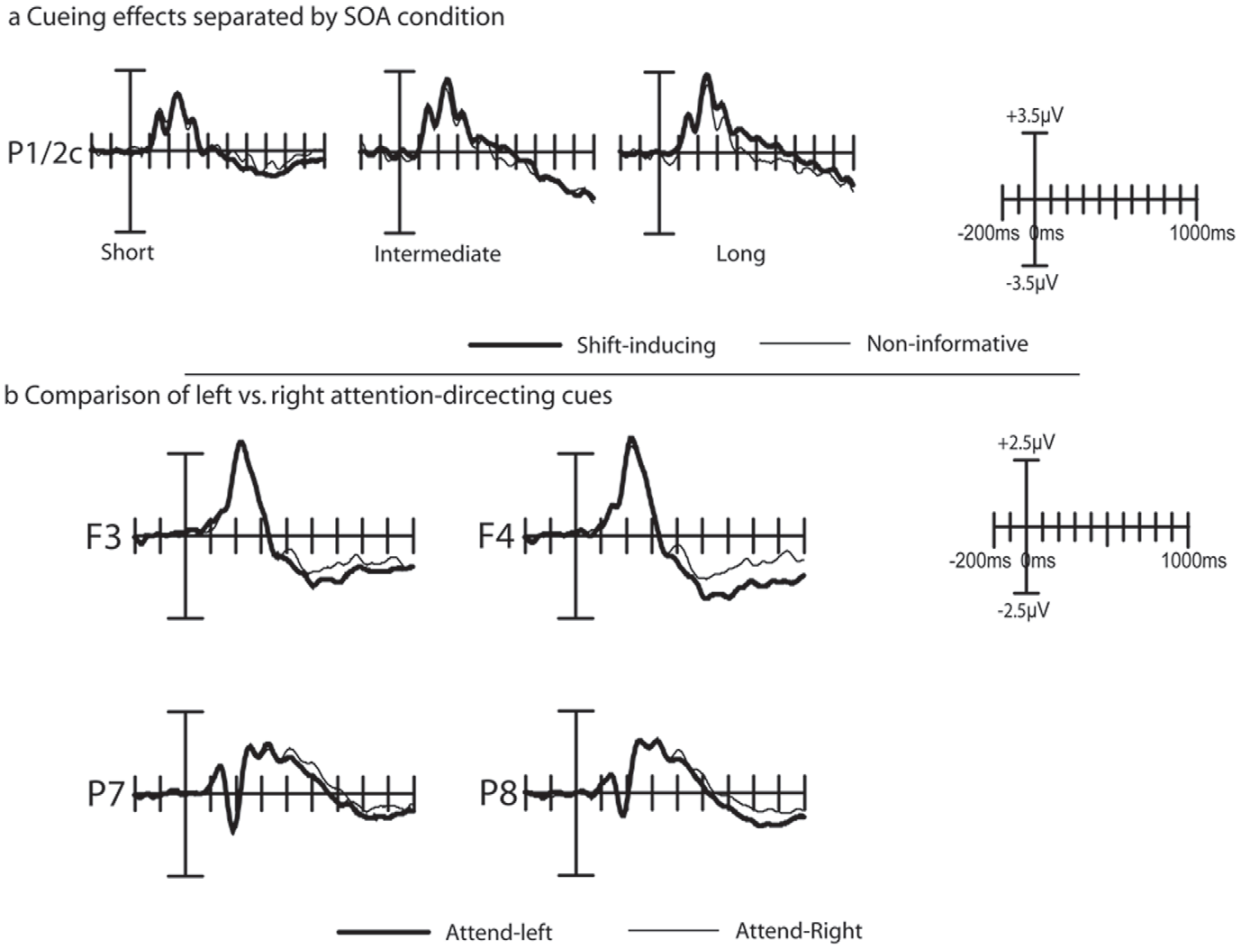
Additional cue-related effects in Experiment 1. (a) Cueing effects as a function of SOA condition. In all three SOA conditions, the shift-inducing cues elicited a similarly enhanced positive deflection within the approximate 250–450 ms time window that has been labeled as shift-related positivity. (b) Comparison of ERPs elicited by left . vs. right attention-shift inducing cues. A small but significant interaction between Cue Direction and Hemisphere between 240 and 280 ms over electrodes P7 and P8 indicates the presence of an EDAN component in these data. The plots in panel (b) are based on left vs. right hemisphere data (i.e. prior to transposing to ipsi- vs. contralateral positions).

Interactions between Cue Type and Difficulty could be observed between 540 and 600 ms, where for cues predicting an easy target a pronounced cueing effect could be observed, whereas this was not case for the cues predicting a hard target (Fs(1,15) = 4.98–8.43; ps<.05-.01). In addition, between 440 and 480 ms after cue onset, a significant 3-way interaction between CueType, Laterality, and Difficulty was significant (Fs(2,30) = 4.69–4.72; ps<.05). This interaction confirms the observation in Figure 4a that the contralateral shift-inducing cues were positively shifting, compared to the non-informative cues, whereas this shift could not be observed for the hard cues. No evidence was found for our hypothesis that the shift-related positivity changed as a function of SOA (see Figure 5a).

Finally, as shown in Figure 5b, a direct comparison between leftward and rightward pointing cues revealed that an interaction between Cue Direction (left vs. right) and Hemisphere (left vs. right) was significant between 260 ms and 300 ms after cue-onset (Fs(1, 15) = 4.53–9.48; ps<.05-.01), when tested over electrodes P04 and P03. When tested over electrodes P7 and P8, this interaction was significant between 240 and 280 ms (Fs(1,15) = 5.36–6.52; ps<.05). Over frontal electrodes, this interaction was not significant; we therefore have no direct evidence for the presence of an ADAN in the present experiment.

### 2.3 Discussion

The goal of this experiment was to investigate the time-course of the processes involved in attentional orienting, as revealed by comparing ERP activity elicited by attention shift-inducing cues against a non-informative, “neutral” baseline condition. In addition we addressed the question how task demands, as reflected in SOA between cue and imperative stimulus and the anticipated difficulty of the cue, affected the attentional shift process. Even though the behavioral results show that responses to validly cued target stimuli were always faster than those to invalidly cued stimuli, the response pattern to non-informative cues gradually changed with increasing SOA. At the longest SOA, the response time to the target following a non-informative cue was in between the response time to a validly and invalidly cued target. At the intermediate and short SOAs, however, the response time to non-informatively cued targets did not differ significantly from that of invalidly cued targets. Therefore these data seem to suggest that it takes longer to process the non-informative cue than it takes to process an informative one. The current pattern of response times are similar to those reported elsewhere [23], in particular those to the difficult targets. In previous behavioral studies (e.g., [11]), response times to non-informative cues were already intermediate (compared to response times to validly and invalidly cued targets) at much shorter SOAs than those reported here. Our current finding is in good agreement, however, with an earlier conclusion that the non-informative cue is somewhat less alerting and results in some additional processing of the imperative stimulus when the SOA between Cue and Imperative Stimulus is short [23]. It should be noted, however, that earlier studies used arrow symbols as cues, which are now known to be somewhat reflexive in nature [28], [29], [30]. Therefore, in the current study the longer time required to establish this pattern could well reflect some additional requirement to process the genuine endogenous cue.

The present data also indicate that the cueing effect could be modulated by the anticipated difficulty of the forthcoming targets stimulus. This was reflected in a significant contralateral difference in cueing effect between the cues predicting easy and cues predicting hard targets that starting at around 440 ms after stimulus onset. It should be noted that the ERP difference between shift-inducing and non-informative cues was not only larger for cues predicting an easy target, but also inverted in polarity from cues predicting an easy target to cues predicting a hard target; whereas on the easy trials the ERPs elicited by shift-inducing cues was more positive, on the hard cues this ERP was more negative, compared to the ERP elicited by the non-informative cues.

This difference in cue-related activity preceding easy and hard targets could possibly reflect the selective difference in the level of preparation or anticipation that is following the cue. Anticipatory processes are typically reflected in a negative slow wave ERP waveform, known as the contingent negative variation (CNV) [31]. The relative increase in the negative potential elicited by the attention-directing cues, from anticipating an easy target to anticipating a difficult target could therefore reflect a significant increase in preparation, in particular when the location of the targets can be anticipated. Although we consider this a plausible interpretation, it should also be noted that the ERP effects we report here are also different from the traditional CNV in several ways. Firstly, the CNV is typically characterized by an early phase that has a fronto-central scalp distribution and a later phase that is characterized by a more central scalp distribution that is thought to represent motor preparation (e.g. [32]). In contrast, the longer latency effects observed in the present study were characterized by a more parietal distribution. Secondly, the CNV is typically characterized by a more medial scalp distribution, which appears to be inconsistent with the current observation that the shift related difference is found mostly over contralateral areas. More recent studies also reported a more posterior contralateral negative slow wave (CNSW) activity related to various cognitive processes involving either preparation or maintenance of information in working memory [33], [34], [35]. This CNSW has been shown to increase in negativity with increasing memory load [35], interacts with selective attention processes [36], and presumably reflects changes in the neural firing pattern of neurons building a visual representation of extrapersonal space.

It is interesting to note that the ERP data show an early dissociation between the attentional directing cues and the non-informative cues that could be related to the somewhat atypical response times at short SOAs. In particular, attention-directing cues elicited a reduced ipsilateral N1 and an enhanced contralateral N1, compared to the non-informative cues. Since we can rule out interpretations in terms of physical differences between the cues, this N1 amplitudes difference is therefore likely to reflect a very early latency change in sensitivity in the occipital brain areas, in response to an attention-directing cue that is not present for the non-informative cues. To our knowledge, this study is the first to report such an early latency cueing effect that cannot be attributed to any underlying sensory difference. The observation that at short SOAs response times to non-informative cues are still somewhat larger than those to the other cue types could be indicative of the fact that attention remains longer focused at the central location in the presence of a non-informative cue. This, in turn, would lead to a longer latency orienting process that could be reflected in the longer latency at which these orienting effects are reflected in the ERP waveform.

To further test the hypothesis that attention indeed remains initially at fixation in response to a non-informative cue, a second experiment was conducted. Here participants were explicitly required to maintain their focus of attention at the center of fixation whenever a non-informative cue was presented. We expected that if the early latency effects observed in Experiment 1 are indeed a reflection of a prolonged dwelling of attention at fixation, then the early latency cueing effects in Experiment 2 should resemble those of Experiment 1. In addition, because we also presented targets at the center location, these stay-central cues indicated with 100% validity that the target would appear at the center. For this condition we expected response times that would be approximately equal response times to validly cued peripheral targets because in both cases we assume that attention would be fully focused on the location indicated by the cue.

### 2.4 Experiment 2

The main goal of this experiment was to isolate the ERP activity related to the shifting of attention. To this end we designed a spatial cueing task in which ERPs evoked by attention-shift-inducing cues could be compared to those evoked by an equally non-ambiguous cue that did not require a shift of attention. We accomplished this by using a cue that was no longer non-informative about the upcoming target location (as in Experiment 1) but instead instructed participants to keep spatial attention focused at the already attended central location. We labeled this cue as the stay-central cue. In contrast to the ambiguous non-informative cues used in Experiment 1 (which are typical for spatial cueing tasks), our stay-central cue is unambiguous because the target stimulus was presented with 100% validity at the central location. In other words, when presented the stay-central cue, participants had every reason to remain focused at the central location. Even though the physical characteristics of these stay-central cues were identical to those of the non-informative cues in Experiment 1, they were functionally different. If a non-informative cue results in participants not shifting attention, we expect to find similar ERPs in the current experiment in which the stay-central cue explicitly instructed participants to keep spatial attention at the center.

### 2.5 Results

#### 2.5.1 Behavioral Data

Mean response times to correctly responded target stimuli are shown in Figure 2b. Response times were larger at the long SOAs, as indicated by a significant main effect of SOA (*F*(2,34) = 20.9; *p*<.00001). In addition, a significant cueing effect was found (*F*(2,34) = 26.0; *p*<.00001). Compared to Experiment 1, the following differences can be noted. First, since targets were presented centrally, no response time differences were obtained between validly cued peripheral targets and the central targets (F<1). Longer reaction times, elicited by invalidly cued peripheral targets were found at each SOA condition, as expressed by a significant response time difference between invalidly cued targets on the one hand and validly (F(1,17) = 56.6; p<.00001) or central targets (F(1,17) = 28.0; p<.0001) on the other hand. No interactions between Cue Type and SOA were found.

Mean hit rate was about 84%. A significant cueing effect on hit rate was observed (F(1,17) = 8.89; p<.0001). Post-hoc testing showed that hit rates did not differ significantly between validly cued peripheral targets and central targets (F(1,17) = 2.04; p<.2). In contrast, hit rates were significantly lower for invalidly cued targets, compared to either validly cued targets (F(1,17) = 11.64; p<.005) or central targets (F(1,17) = 11.89; p<.005).

#### 2.5.2 ERP data

As shown in Figures 3b and 4b, the cue symbols elicited a well-established sequence of ERP components that, in general, resembled those obtained in Experiment 1. Over parietal and occipital areas, P1 and N1 components can be observed, that are followed by more broadly distributed P2 and N2 components, which are followed by a sustained negative sloping potential at longer latencies. It should be noted that the amplitude of the early P1 component, elicited both by the shift-inducting cues and the stay-tuned cues, were somewhat larger than the P1 components obtained in Experiment 1, and that consequently the entire cue-elicited wave form was shifted positively.

The earliest significant effect in this experiment constituted the P1 component (Figure 3b). The P1 effect was the most pronounced over the hemisphere ipsilateral to the direction of the shift inducing cue. This P1 enhancement was reflected in a significant interaction between Cue Type and Hemisphere, when tested using mean voltages between 130 and 140 ms from electrodes P3/4i and P3/4c as input (F(1,17) = 4.89; p<.05). The P1 effect was followed by a cue related contralateral enhancement of the N1 component, which was also reflected in a significant interaction between cue-type and hemisphere (F(1,17) = 4.62; p<.05).

Finally, Figure 4b suggests the presence of an early negative deflection elicited by the stay-central cues, at posterior electrodes P1,Pz, P2, PO1, POz, and PO2, at around 80 ms after cue onset, particularly so for the cues predicting an easy target. Although no main effect of Cue Type was found in this latency range, the interaction between Cue Type and Difficulty was significant between 60 and 120 ms (Fs(2,34) = 4.91–10.31; ps<.05-.005).

Figure 4b shows the time course of the longer latency cueing effects. Some notable differences between these results and those of Experiment 1 can be noticed. First, the shift-related positivity that was observed in Experiment 1 can no longer be seen, and consequently, no significant main effects of cue type could be observed in this time window. A second major difference between these data and those from Experiment 1 is that here the ERPs elicited by the stay-central cues are shifting positively at around 500 ms after cue onset, compared to the shift-inducing cues, particularly so for the cues that predicted and easy target. This effect was reflected in a three way interaction between Cue Type and Difficulty that was significant between 660 and 1000 ms after cue onset (Fs(1,17) = 7.48–53.5; ps<.05 - .00001). No further effects were found significant.

**Figure 6:**
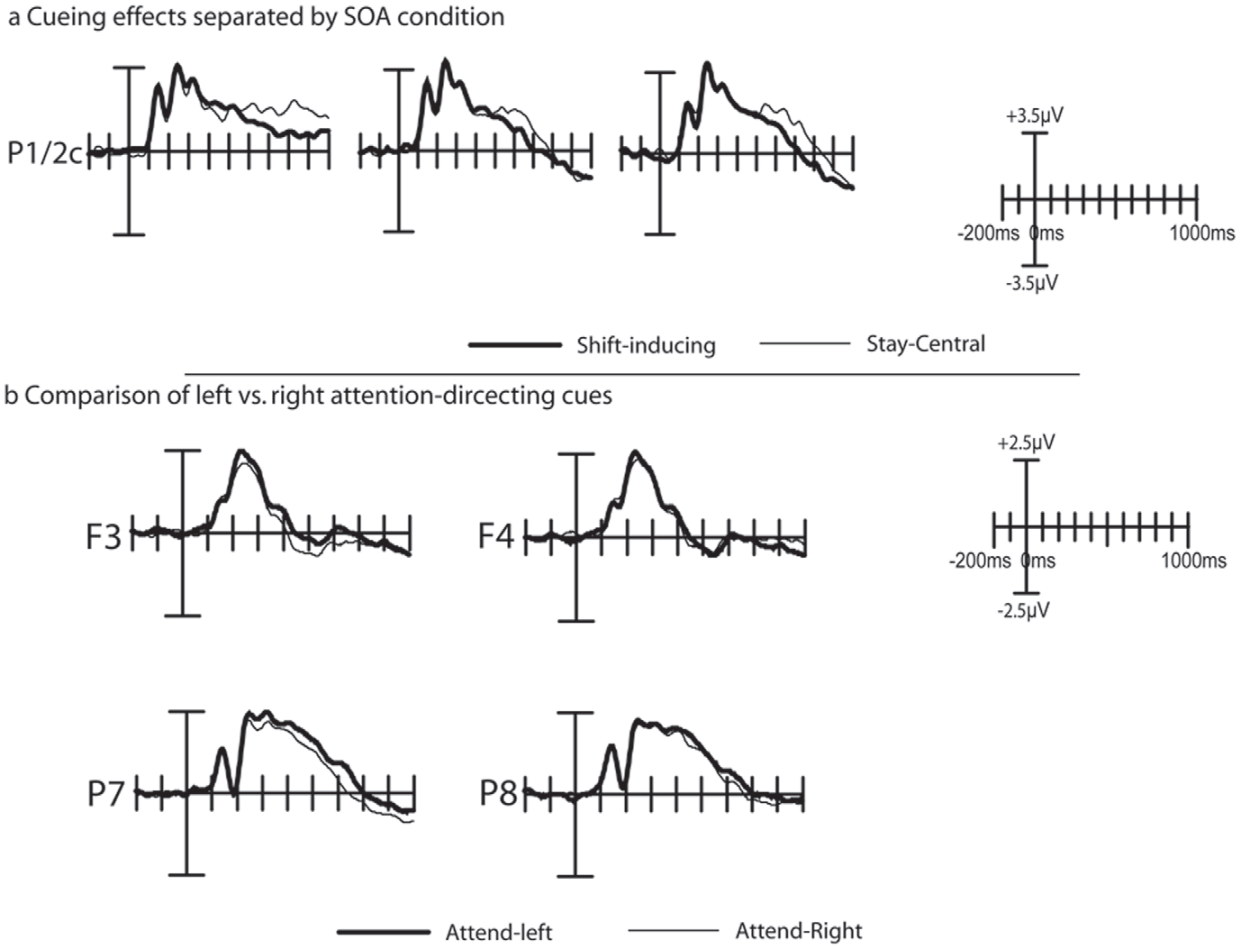
Additional cue-related effects in Experiment 2. (a) Cueing effects as a function of SOA condition. In all three SOA conditions, the shift-inducing and stay-central cues elicited similar positive deflections within the approximate 250–450 ms time window indicating that the shift-related positivity was no longer present in experiment 2, and these components were similar across SOA conditions. (b) Comparison of ERPs elicited by left . vs. right attention-shift inducing cues. Significant interaction between Cue Direction and Hemisphere over electrodes P7 and P8 indicate the presence of an EDAN component in these data. In addition, with a somewhat later onset, an ADAN component can also be observed. The plots in panel (b) are based on left vs. right hemisphere data (i.e. prior to transposing to ipsi- vs. contralateral positions).

Finally, as shown in Figure 6b, a direct comparison between leftward and rightward pointing cues revealed that an interaction between Cue Direction (left vs. right) and Hemisphere (left vs. right) was significant at electrodes P7 and P8 between 280 ms and 340 ms after cue-onset (Fs(1, 17) = 4.30–6.99; ps<.05). No significant interaction could be observed on electrodes PO3 and PO4 in this latency range. Over frontal electrodes F3 and F4, a significant interaction between the factors Cue Type and Hemisphere became slightly later significant; between 460 and 680 ms after cue onset (Fs(1,17) = 4.26–13.10; ps<.05 - .005).

### 2.6 Discussion

This experiment was designed to evaluate processes involved in shifting vs. not shifting attention. Because our “stay-central cue” and valid peripheral cue both indicated the location of the upcoming target we expected to find response times in these two conditions which would be approximately the same. Our behavioral data are consistent with this notion, because the response times to valid central and valid peripheral targets were not significantly different from each other. The latter result should be interpreted with some caution, because the target stimuli were presented at the central location in the “stay-central” condition, whereas they were presented at peripheral locations in the attention-shift conditions. However, because the targets presented at central locations were adjusted to be approximately equally difficult to those presented at the peripheral locations, we do argue that the deviant reaction time to the invalid cue represents mainly the costs of reorienting to the opposite location. This suggests that attention had to be shifted from the location indicated by the cue to the location where the target was located.

We expected that if the early latency effects observed in Experiment 1 are indeed a reflection of a prolonged dwelling of attention at fixation, then the early latency cueing effects in Experiment 2 should resemble those of Experiment 1. In line with this expectation, shift-inducing cues elicited larger contralateral and smaller ipsilateral N1 components, than the stay-central cues. These results corroborate findings from Experiment 1, that an early latency shift in occipital responsiveness can be induced by a purely symbolic cue.

It should be noted that some additional early latency results were found in Experiment 2 that were not present in Experiment 1. First, in Experiment 2, the N1 amplitude effect was preceded by a parietal P1 effect over the ipsilateral hemisphere. Here, the P1 amplitude was larger in response to the shift-inducting cue then it was to the stay-central cue. Because the P1 component has been interpreted as being sensitive to the suppression of irrelevant locations [23], [24], [37], this component could reflect an initial selection process related to the suppression of locations that are known to be irrelevant. The early latency of this effect is remarkable, because it suggests that the meaning of the cue can be extracted within approximately the first 150 ms after cue presentation. Although the early occurrence of this effect seems rather remarkable, the latency of this P1 effect is in fact consistent with recent work showing that people are able to reliably make a saccade to the side of a natural scene that contains an animal in as little as 120 ms [38], suggesting that the gist of a scene can be extracted extremely fast. Although clearly more research is needed, we propose that a similar mechanism might underlie the extraction of the gist of the cue-symbol meaning in the present study.

Secondly, a highly significant early latency interaction between cueing and anticipated target difficulty was observed, that only trended toward significance in Experiment 1. Although we are currently unsure what this effect represents, it could be taken as an indication that an attentional shift could be initiated more rapidly when the subsequent target stimulus is expected to be easy. Finally, the shift-related positivity observed in Experiment 1 was no longer present in this experiment, suggesting that this process is more likely to represent processes other than the active shifting of attention in response to a shift-inducing cue.

## Discussion

The present study was designed to address the question to which extend a non-informative cue can be used as a neutral baseline condition to isolate attentional orienting processes. In addition, we investigated the degree to which electrophysiological reflections of attentional orienting processes can be influenced by the amount of time that is given to fully orient attention. To rule out the possibility of attributing our observations to physical differences between cues, we used letters as attention-directing cues. The present study reveals several important novel effects: an early latency cuing effect; longer latency effects of cue difficulty, and a marked effect of using a stay-central cue opposed to using a non-informative cue. We will discuss each of these effects below.

### 3.1 Early latency effects

Clearly the most novel finding of this study consisted of an early latency N1 effect that was observed in response to a shift inducing cue. Although N1 amplitude effects are commonly associated with attention, these effects are usually observed in response to a target stimulus that is presented at the attended location [23], [24], [26], [37]. However, no such early latency attention effects have been reported in response to the cue symbol itself. Although N1 amplitude enhancements have been reported previously [23], [24], [26], these earlier studies used physically different cue symbols, and could therefore not unequivocally attribute these results to an attentional task parameter. Since this effect was replicated across the two experiments presented here, we propose that this N1 effect is a genuine reflection of an early latency process involved in attentional orienting. Although the exact functional meaning of this effect is not immediately clear, we propose that it is related to the process of segregating the shift inducing cues from either the non-informative (Experiment 1) or stay-central (Experiment 2) cues ([38]).

Longer latency differences in cueing effects between across the two experiments suggest that attention does not necessarily remain at fixation when a non-informative cue is used. In particular, the 200–400 ms latency effect that we previously labeled as the “shift-related positivity” could not be found in Experiment 2. As indicated by the behavioral data, in both experiments attention was shifted efficiently to the peripheral locations in response to the shift inducing cue. Therefore, the main reason for the diminishing of this positive shift has to be attributed to the fact that the non-informative and “stay-central” cues evoke different processes in the 200 to 400 ms time window. As discussed below, we interpret the difference in cueing effect across the two experiments to reflect a difference in the information content that is provided by the cue types.

### 3.2 Non-Informative vs. Stay-Central Cues

The second novel finding indicates that ERP responses differ markedly between non-informative cues (experiment 1) and stay-central cues (experiment 2). More specifically, we found in experiment 1 a positive deflection between about 200 and 400 ms, elicited by shift-inducing cues, that we had previously labeled as “shift-related positivity” [24]. The fact that this positive difference is largely absent in experiment 2, shows that this effect cannot be related to the shifting of attention itself, but presumably represents something else. One possibility is that this positive difference reflects activation of the neural generators of the P300 component. The P300 component is known to be sensitive to the information content of the eliciting stimulus [39], [40]. More specifically, these studies showed that the P300 component increases in amplitude when a cue carries more information. Consistent with Gratton et al. 's results we found that in experiment 1, the more meaningful attention-shift cues elicited a larger P300 component than the non-informative cue. In contrast, no such difference was found in experiment 2, where the attention-shift cues were equal in information content as the stay-central cues. This interpretation is also consistent with data from Slagter and colleagues [41], [42], who showed that symbolic cues elicited a larger P300-like component when the cue are presented in a context where they are more meaningful, as to when they are presented in a less meaningful context.

### 3.3 Difficulty Cueing

The present results show that participants can selectively anticipate the difficulty of an upcoming target stimulus. When the cue indicated that the upcoming target stimulus would be easy to detect, ERP effects were generally larger than when these cues indicated that the targets would be hard to detect. This result is somewhat contrary to our hypothesis, because we had expected that anticipating a difficult target would result in a stronger focusing of attention. The significant interactions between cueing and difficulty, occurred at somewhat longer latencies, and correspond to a positive shift in the ERP waveform, that occurs at around 400 ms (Experiment 1) to 500 ms (Experiment 2). Of the cues instructing to anticipate an easy target, in Experiment 1 it is the shift-inducing cue that shows this positivity shift, whereas in Experiment 2, it is the stay-central cue that shows this positive shift. One possibly simple explanation for this seemingly complex pattern is that this positive shift reflects the strategic allocation of attention under conditions when a critical level of confidence can be reached. In Experiment 2, the stay-central cue is more predictive than the shift inducing cue, whereas the non-informative cue of Experiment 1 is less predictive than the shift inducing cue. Thus, it is likely that relatively less preparatory processes are allocated in case of a non-ambiguous situation, which could explain the marked reduction of CNSW activity in the stay-central condition; specifically when an easy target is expected. Although this interpretation is still somewhat speculative, previous studies have established a relation between a positive ERP shift and confidence in memory processes [43], [44], found an inverse relation between a negative going shift and familiarity of a recognized item. Even though these processes are somewhat different from the attention shifts reported in the present study, we argue that a common mechanism might be underlying these observations.

### 3.4. Summary and conclusions

This study investigated the mechanisms of shifting attention, and the role of task demands on these processes, and it validated the use of a non-informative cue in symbolic cueing paradigms. We found a significant N1 amplitude modulation that we interpret as an early latency attentional biasing process. Since this effect was similar across the two experiments, we conclude that attention initially remains at fixation in response to a non-informative cue. In contrast, we observed a subsequent cueing effect in experiment 1 (∼200–400 ms post-cue) that was largely absent in experiment 2. This effect, which we previously interpreted as a “shift-related positivity”, is presumably driven by the information content of the cue. In addition, we found a longer latency positive shift that appeared to be inversely related to difficulty and stimulus ambiguity. We conclude that this component reflects the engagement of attention under conditions of relative confidence about the anticipated difficulty of the target and high predictability of the upcoming target location.

## Materials and Methods

### 4.1 Ethics Statement

Written consent was obtained from each participant prior to the experiments. The experiments were approved by the local ethics committee of the Vrije Universteit, Amsterdam and adhered to the declaration of Helsinki.

### 4.2 Experiment 1

#### 4.2.1 Participants

Eighteen healthy volunteers participated in the present study (mean age: 20.6; range 18–34; 11 females). Two participants were excluded from the analysis, because they were not able to comply with fixation instructions. Participants were recruited through local on-campus advertisements. All participants had normal or corrected-to-normal vision. None of them reported a history of mental or physical illness. All participants received a financial compensation.

#### 4.2.2 Stimulus presentation and design

Three white outlined squares were presented on a black background of a computer screen, with a white fixation dot in the middle of the central square (see Figure 1). The squares subtended a vertical and horizontal angle of 1.5°. The peripherally located squares were centered 3.6° left or right and 4.0° below fixation. Each trial started with a symbolic cue, which was followed by an imperative stimulus. The symbolic cue was presented inside the central square for 100 ms. In 33% of the trials the symbolic cue was non-informative, whereas in the remaining 67% of the cases, the cue instructed to attend to one of the two peripherally located squares (shift-inducing cue). On 80% of the shift-inducing cue trials, the cue correctly indicated the location of the following imperative cue (valid cue), whereas on 20% of the attention-directing cues, the opposite peripheral location was cued (invalid cue). In all trials the cue correctly indicated the difficulty (easy, hard) of the following imperative stimulus, which allowed participants to selectively prepare for the difficulty of this stimulus.

The symbolic cue consisted of one of the characters a, b, or c in a red or blue color, and subtended a vertical angle of about 1.1°. The “URW Gothic” font type was used, because in this font the “a”, “b”, and “c” characters are very similar in appearance. Each character represented a location and each color represented the level of difficulty following the cue. The specific cue-symbol mapping was counterbalanced across participants. For example, for participant 1, the mapping was: a = Left, b = Non-Informative, c = Right, Blue = Easy and Red = Hard while for participant 6 it was a = Non-Informative, b = Right, c = Left, Blue = Hard and Red = Easy (see Figure 1b for an illustration of the cue-symbol mapping). After an SOA of 200–800 ms (short), 800–1600 ms (intermediate) or 1600–2400 ms (long), the imperative stimulus was presented, which consisted of a brightening of the outer lines of one of the two peripheral squares. 20% of the imperative stimuli were designated as targets. Targets were characterized by the presentation of an additional faint grey dot in either the lower left or lower right corner of the brightened square [45]. The contrast between this dot and the background was low on the ‘hard’ trials while it was higher on the ‘easy’ trials (gray values: hard = 0.412 cd/m^2^ to 8.639 cd/m^2^, easy = 13.37 cd/m^2^ to 39.85 cd/m^2^, background = 0 cd/m^2^). Within the two levels of difficulty, faintness was randomly varied on a trial-to-trial basis. Easy and hard target trials were randomized with equal probability. Short, intermediate, and long SOA trials were presented in a block-wise fashion, and counterbalanced among participants.

#### 4.2.3 Procedure

Participants were instructed to direct and focus their attention to the location instructed by the cue. Thus, in case of a shift-inducing cue, participants had to shift their attention to one of the two peripheral locations. In case of the non-informative cue they were required to stay focused centrally. Participants were instructed to respond to the target stimulus as fast as possible, regardless of whether the target appeared at a cued or uncued location. Responses were made by pressing a response button, using the index finger of the participant's preferred hand. Participants were further instructed to keep their eyes fixated on a dot placed at the center of the screen and to avoid blinking as much as possible. Before the start of the session, participants were given the opportunity to practice the task until the experimenter was convinced they understood the task.

The experiment consisted of 9 blocks of 140 trials each. Each session consisted of 3 blocks with a short SOA, 3 blocks with an intermediate SOA and 3 blocks with a long SOA. The length of the blocks varied between 6 (short SOA) and 10 minutes (long SOA). For each participant and block of trials, a new randomized trial sequence was generated.

Between blocks, participants were given a short break. The total length of the session (excluding preparation and instruction) was approximately 90 minutes.

#### 4.2.4 Instrumentation and recording

Stimulus presentation was controlled by an Intel Pentium 4 personal computer, running the Windows XP operating system. Stimuli were presented on a 17″ CRT screen using E-prime software (Psychology Software Tools, Inc., Pittsburgh, PA). The screen was located at a distance of 135 cm from the participant. Electroencephalographic (EEG) signals were recorded in DC mode (i.e. without using a high-pass filter) using 60 tin electrodes mounted in an elastic cap (Oz, O1, O2, POz, PO1, PO2, PO3, PO4, PO5, PO6, Pz, P1, P2, P3, P4, P5, P6, P7, P8, CPz, CP1, CP2, CP3, CP4, CP5, CP6, CP7, CP8, C1, C2, C3, C4, C5, C6, C7, C8, FCz, FC1, FC2, FC3, FC4, FC5, FC6, Fz, F1, F2, F3, F4, F5, F6, F7, F8, AFz, AF1, AF2, FPz, FP1 and FP2), referenced against the right-mastoid. Horizontal and vertical eye movements were measured using bipolar recordings from electrodes placed on the outer canthi of the two eyes and from electrodes placed approximately 1 cm above and below the participant's right eye. Electrode impedance was kept below 5 kΩ. All EEG data were recorded at a sample frequency of 1000 Hz, using two Neuroscan SynAmps amplifiers (Compumedics Ltd Corporate, El Paso, TX) connected to a second Intel Pentium 4 personal computer, running Neuroscan's “scan” software version 4.2. EEG data were digitally stored for offline analysis.

#### 4.2.5 Data-analysis

Mean response times to correctly reported targets were calculated for each participant. This was done separately for each cue type, and also separately for the three SOA conditions. Outcomes were statistically analyzed using repeated-measures Analysis of Variance (ANOVA) containing the factors Cue Validity (valid, non-informative, or invalid), and SOA (short, intermediate, or long) as within-subjects factors.

Likewise, for each participant, hit-rates to target stimuli were calculated separately for each cue-type and SOA condition. These values were also subjected to a repeated measures ANOVA, containing the factors Cue Validity (valid, non-informative, or invalid), and SOA (short, intermediate, or long) as within-subjects factors. In all tests, Greenhouse-Geisser (GG) correction was applied where necessary.

Response times were larger and hit rates were significantly lower to difficult targets than to easy targets. Since this difference could be ascribed to the physical differences between these stimuli, as opposed to the experimental manipulations of interest, and because the behavioral effects on target difficulty did not interact with any of the other factors, all behavioral data were collapsed across these two conditions.

Trials containing eye movement related amplitude fluctuations exceeding +/−50 µV/100 ms as well as trials containing DC offset jumps (exceeding 1000 µV/5 ms due to manual DC offset corrections) were excluded from the averaging procedure. Blink artifacts were corrected using a regression method [46]. EEGs were filtered using a 4096 point half-Gaussian finite impulse response (FIR) high-pass filter at 0.06 Hz. and a 128 point half-Gaussian FIR low-pass filter at 25 Hz. Condition-wise ERP averages were then computed for cues and imperative stimuli. Any remaining artifacts in the EEG, i.e. those not related to ocular movements of DC offset corrections were detected during an auto-adaptive procedure that automatically excludes artifact-bearing trials [47]. These resulting averages were corrected for possible overlap between cue and target ERPs using the adjacent response (ADJAR) method [48]. After averaging and overlap correction, ERPs elicited by left and right shift-inducing cues were collapsed in to a single waveform. This was done by rearranging all the data in the shift right condition such that all the left hemisphere electrode locations were transposed to the right hemisphere, and vice versa. Then, data from the thus transposed shift right condition was combined with that of the original shift left condition (cf. [24], [49]).

Early latency cue-elicited ERP components were tested for possible attention effects by collapsing the ERP waveforms across the factors Difficulty, and SOA, and subjecting mean voltages surrounding the early P1 (130–140 ms after cue onset) and N1 components (180–200 ms after cue onset) to ANOVA containing the within-subject factors Cue Type (two levels: Shift-inducing vs. Non-Informative), Hemisphere (two levels: Ipsilateral vs. Contralateral), and Electrode (three levels: O1/2, PO3/4, and PO7/8). These electrode locations are consistent with the electrode locations that we used in earlier reports for the analysis of early latency P1 and N1 modulations [23], [24].

The longer-latency cue-elicited ERP effects were statistically tested by computing mean amplitudes in consecutive time windows of 20 ms, at electrodes C1, Cz, C2, CP1, CPz, CP2, P1, Pz, and P2. These electrodes were chosen on the basis of prior visual inspection of the data, which revealed that attention effects were the most pronounced over these areas. Mean voltages were submitted to repeated-measurement ANOVAs, containing the within-subject factors of Cue Type (shift-inducing vs. non-informative), SOA (short, intermediate or long), Difficulty (hard or easy), Laterality (ipsilateral, midline, or contralateral hemisphere electrodes), and Area (central, centro-parietal, or parietal). Only effects between 0 and 1000 ms after cue-onset are reported, as previous research has suggested that shift-related processes are usually finished by that time [50].

Finally, the ERPs elicited by leftward vs. rightward attention-directing cues were contrasted, using a similar approach as described above. Here, the original left and right hemisphere data were used as input for a within-subjects ANOVA containing the factors Hemisphere (left vs. right), and Cue Direction (left vs. right). This analysis was run separately for electrode pairs PO3 and PO4, P7 and P7 to test for the presence of an EDAN, and electrode pair F3 and F4 to test for the presence of an ADAN. These electrode locations were chosen on the basis of our previous work [23], which used similar electrode positions for this type of analysis.

### 4.3 Experiment 2

#### 4.3.1 Participants

An additional eighteen healthy volunteers participated in the present study (mean age: 22.1; range 18–31; 14 females). Inclusion criteria and procedures were the same as those for Experiment 1.

#### 4.3.2 Stimulus presentation and design

The design of this experiment was identical to that of Experiment 1, with the following exception: Whereas in Experiment 1 the non-informative cue was followed by an imperative stimulus that was randomly presented in the left or right hemifield, here this cue type was always followed by an imperative stimulus that was presented at the central location (i.e. a brightening of the central box surrounding the fixation cross. In other words, the stay-central cue was always 100% valid. Since target stimuli were now present near fixation is this condition, the brightness of the target stimulus presented in the central box was adjusted to compensate for greater acuity near fixation, thus equating task difficulty between the stay-central and attention-shift conditions as much as possible. Brightness values for these centrally presented targets were adjusted using pilot studies, and were in the range of 1.0 cd/m^2^–6.291- cd/m^2^ (hard target) and 8.639 cd/m^2^ to 34.81 cd/m^2^ (easy target).

#### 4.3.3 Procedure

The procedure for this experiment was also identical to that of Experiment 1, except that participants were explained that the stay-central cue would be followed by a brightening of the central box. Also, target stimuli following the stay-central cue would be presented in the lower left or right corner of the brightened central box. As in Experiment 1, it was emphasized that participants should keep attention centrally when this type of cue was presented. All other aspects of this experiment (equipment, recording procedures, and analyses) were identical to that of Experiment 1.
